# The Role of Herbivory in Structuring Tropical Seagrass Ecosystem Service Delivery

**DOI:** 10.3389/fpls.2018.00127

**Published:** 2018-02-12

**Authors:** Abigail L. Scott, Paul H. York, Clare Duncan, Peter I. Macreadie, Rod M. Connolly, Megan T. Ellis, Jessie C. Jarvis, Kristin I. Jinks, Helene Marsh, Michael A. Rasheed

**Affiliations:** ^1^Centre for Tropical Water and Aquatic Ecosystem Research, James Cook University, Cairns, QLD, Australia; ^2^College of Science and Engineering, James Cook University, Townsville, QLD, Australia; ^3^Centre for Integrative Ecology, School of Life and Environmental Sciences, Deakin University, Burwood, VIC, Australia; ^4^Australian Rivers Institute-Coast and Estuaries, School of Environment, Griffith University, Nathan, QLD, Australia; ^5^Gladstone Ports Corporation, Gladstone, QLD, Australia; ^6^Department of Biology and Marine Biology, Center for Marine Science, University of North Carolina Wilmington, Wilmington, NC, United States

**Keywords:** grazing, herbivore, seagrass, ecosystem services, dugong, turtle, fish, mesograzer

## Abstract

Seagrass meadows support key ecosystem services, via provision of food directly for herbivores, and indirectly to their predators. The importance of herbivores in seagrass meadows has been well-documented, but the links between food webs and ecosystem services in seagrass meadows have not previously been made explicit. Herbivores interact with ecosystem services – including carbon sequestration, cultural values, and coastal protection. Interactions can be positive or negative and depend on a range of factors including the herbivore identity and the grazing type and intensity. There can be unintended consequences from management actions based on a poor understanding of trade-offs that occur with complex seagrass-herbivore interactions. Tropical seagrass meadows support a diversity of grazers spanning the meso-, macro-, and megaherbivore scales. We present a conceptual model to describe how multiple ecosystem services are influenced by herbivore pressure in tropical seagrass meadows. Our model suggests that a balanced ecosystem, incorporating both seagrass and herbivore diversity, is likely to sustain the broadest range of ecosystem services. Our framework suggests the pathway to achieve desired ecosystem services outcomes requires knowledge on four key areas: (1) how size classes of herbivores interact to structure seagrass; (2) desired community and management values; (3) seagrass responses to top–down and bottom–up controls; (4) the pathway from intermediate to final ecosystem services and human benefits. We suggest research should be directed to these areas. Herbivory is a major structuring influence in tropical seagrass systems and needs to be considered for effective management of these critical habitats and their services.

## Introduction

Herbivores can dramatically influence primary production through top–down regulation in global ecosystems, including seagrass meadows. Seagrasses are well-adapted to cope with grazing pressure (Heck and Valentine, 2006); however, plant–herbivore interactions can modify characteristics such as biomass, productivity, and species diversity. There are 31 tropical seagrass species, approximately half of the global total, grazed by a broad suite of herbivores (Carruthers et al., 2002; Short et al., 2007). This diversity leads to complex interactions among plants and herbivores. In the tropics, how these interactions shape seagrass meadow properties is not fully understood (York et al., 2017). Such grazer-mediated changes in meadow structure can also influence the ecosystem services provided by seagrass, an area that has received little research focus (Bakker et al., 2016).

The Millennium Ecosystem Assessment outlined four categories of ecosystem services: provisioning, regulating, cultural, and supporting (Millennium Ecosystem Assessment, 2005). These categories have been refined to better reflect how humans use ecosystems and to distinguish between intermediate ecosystem services, final ecosystem services, and benefits (Mace et al., 2012). This new classification prevents double-counting of services in management or economic valuations (Boyd and Banzhaf, 2007; Fisher et al., 2008). Final ecosystem services are ‘aspects of ecosystems utilized (actively or passively) to produce human well-being,’ whereas intermediate services are not used by humanity, either directly or indirectly (Fisher et al., 2008). Benefits are the ways human well-being is enhanced through ecosystem services (Mace et al., 2012), and sometimes require human inputs such as people, knowledge, or equipment (Fisher et al., 2008). Seagrass meadows provide numerous intermediate and final ecosystem services (Nordlund et al., 2016). For example, nutrient cycling in seagrass meadows is an intermediate service, which produces the final ecosystem service of improved water quality, with the benefit of improved human health. Herbivory has the potential to modify these seagrass ecosystem services by reducing biomass, changing productivity, or altering species assemblages within meadows.

**FIGURE 1:**
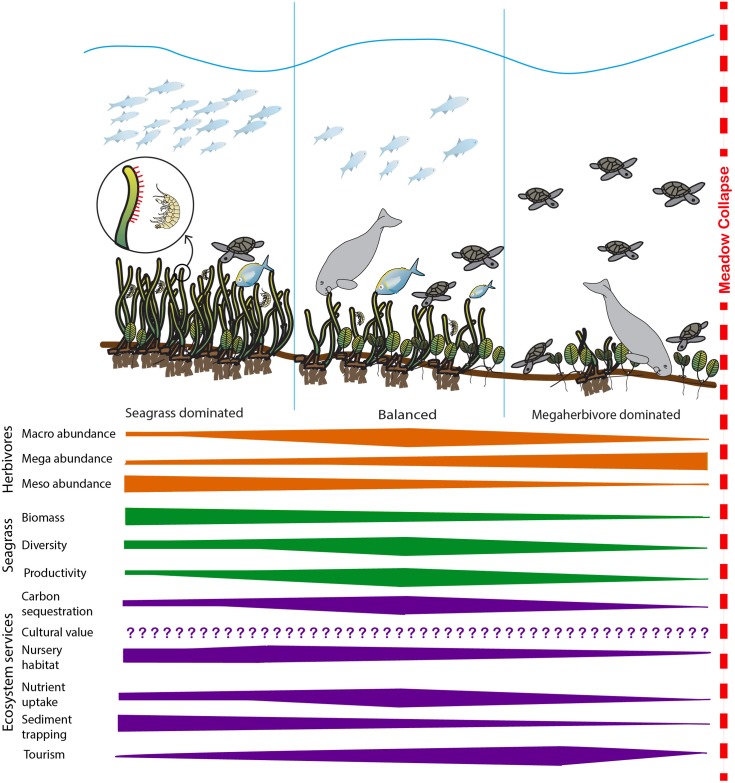
Summary of the expected change in herbivore abundance, key seagrass meadow properties and selected ecosystem services as habitats shift from seagrass-dominated to megaherbivore-dominated. At low levels of herbivory, disturbance is minimal and seagrass biomass dominates the system. As herbivory increases, the system moves toward a balanced state where productivity increases in response to herbivory and productivity-associated ecosystem services’ (i.e., carbon sequestration and storage, nutrient uptake leading to improved water quality) delivery increases. In this system, the diversity of both seagrass and herbivore assemblages are generally at their highest. As herbivory increases further, seagrass biomass, diversity and productivity decreases and most ecosystem services’ delivery reduces before the meadow becomes overgrazed and collapses, at which point ecosystem services’ delivery ceases. Cultural ecosystem services’ delivery may be influenced by herbivory, but responses will be highly variable and changes in cultural ecosystem service delivery with increasing herbivory cannot be confidently predicted (Díaz et al., 2006; Garcia Rodrigues et al., 2017). Bars illustrate likely direction of change and do not signify predicted linear relationships. Images: Catherine Collier, Diana Kleine, Tracey Saxby and Dieter Tracey Integration and Application Network, University of Maryland Center for Environmental Science (http://ian.umces.edu/imagelibrary/).

The multiple ecosystem services provided by seagrass meadows respond to environmental pressure and interact in complex ways, presenting challenges for managers. Science-based management requires knowledge of the trade-offs that arise from antagonistic interactions between ecosystem services. Trade-offs occur when one service is enhanced at a cost to another, and are a common outcome of management decisions, often unrecognized (Raudsepp-Hearne et al., 2010; Bas Ventin et al., 2015). Synergistic interactions occur when the combined effect of ecosystem service responses is greater than the sum of the individual effects, positive and negative (Côté et al., 2016). We contend that an understanding of how herbivores can structure tropical seagrass meadows (see **Figure 1**) is essential for effective management and conservation.

In this article, we review the current literature and identify the plant–herbivore interactions that structure tropical seagrass meadows. We synthesize this information to develop a conceptual model of how seagrass and herbivory interact to deliver ecosystem services. We suggest a management framework to ensure a holistic approach to achieve desired community and management outcomes for seagrasses, herbivores and the ecosystem services they deliver.

## Plant–Herbivore Interactions Structuring Tropical Seagrass Meadows

Herbivores in tropical seagrass meadows are diverse, with a range of feeding strategies, each influencing meadows differently. We classify them into three groups based on size: mesograzers, macroherbivores, and megaherbivores. Mesograzers (e.g., amphipods, isopods, and gastropods) live on seagrass blades, and mainly consume epiphytes (Duffy et al., 2003), although they can also consume seagrass (e.g., Rossini et al., 2014). Macroherbivores (e.g., sea urchins and fish) shred or take bites out of the seagrass blades (Alcoverro and Mariani, 2004). In contrast, megaherbivores, green turtles and dugongs, crop leaves. Dugongs also excavate whole seagrass plants (turtles only excavate in extreme cases) (Marsh et al., 1982, 2011; Christianen et al., 2014). Each herbivore group contributes to structuring seagrass meadows in different ways, influencing biomass, productivity, leaf nutritional quality, species assemblage structure, and meadow extent.

The impact of herbivory on seagrass biomass changes with herbivore size and density. Megaherbivores and macroherbivores can consume significant amounts of seagrass, resulting in biomass declines, particularly when they are present in large numbers (Masini et al., 2001; Fourqurean et al., 2010; Lal et al., 2010; Vonk et al., 2015). In multi-species tropical meadows, biomass declines may only be observed in some seagrass species (Armitage and Fourqurean, 2006). Grazing by fish can result in bare strips, or halos, around reefs (Randall, 1965), and can outstrip production in tropical meadows (Unsworth et al., 2007). Biomass losses from increased megaherbivore and macroherbivore grazing, or high numbers of herbivores, are often accompanied by reductions in shoot density (Preen, 1995; Lal et al., 2010; Burkholder et al., 2013; Bessey et al., 2016), although not always (Moran and Bjorndal, 2005; Mutchler and Hoffman, 2017). Other structural properties including canopy height, leaf width and area, might decrease due to megaherbivore and macroherbivore grazing (Moran and Bjorndal, 2005; Kuiper-Linley et al., 2007; Lal et al., 2010; Ebrahim et al., 2014). In contrast, herbivory by mesograzers can have positive effects on seagrass biomass. These animals feed on leaf epiphytes, which benefits seagrasses by reducing shading (Orth and Van Montfrans, 1984; Reynolds et al., 2014). Experiments show that mesograzers substantially reduce seagrass epiphytes in temperate and subtropical systems (Cook et al., 2011; Whalen et al., 2013; McSkimming et al., 2015) and their presence can increase seagrass biomass (Myers and Heck, 2013).

Herbivory directly affects seagrass productivity, with impacts caused by grazing intensity and the size class of herbivores. Increased productivity has been recorded in response to grazing by megaherbivores (Aragones and Marsh, 2000; Christianen et al., 2012), but when grazing reaches high levels, productivity declines (Fourqurean et al., 2010; Kelkar et al., 2013). We know less about smaller herbivores in tropical meadows, but temperate studies have shown that macroherbivore grazing increases seagrass growth up to a certain point, after which it declines (Vergés et al., 2008) and mesograzer studies show seagrass productivity increases with increasing grazing on epiphytes (Jaschinski and Sommer, 2008). Megaherbivore grazing can also cause the redistribution of productivity within tropical seagrasses, leading to higher leaf growth relative to rhizome growth (Aragones and Marsh, 2000).

Grazing activity can also affect the seagrass species assemblage. Megaherbivore grazing disturbance creates an environment that favors colonizing seagrass species (sensu Kilminster et al., 2015), causing the seagrass meadow to shift toward a grazing-tolerant, early successional stage community (Preen, 1995; Aragones and Marsh, 2000; Kuiper-Linley et al., 2007; Kelkar et al., 2013). The opposite pattern has also been observed where urchins prefer colonizing species, and their grazing maintains the climax community (Vonk et al., 2008). Seagrass diversity increases as meadows recover from disturbance because a mix of climax and colonizer species are present (Rasheed, 2004). Recovery from grazing can take less than a month to years, depending on the grazing intensity and the life history traits of the seagrass species (Aragones and Marsh, 2000; Kilminster et al., 2015; Lefebvre et al., 2017). High herbivore diversity can enhance secondary production in temperate seagrass meadows (Duffy et al., 2003), however, these relationships require more investigation in diverse tropical systems (Clarke et al., 2017).

Herbivores can also have large-scale positive impacts on seagrass meadows: by dispersing seagrass propagules and seeds up to 100s of kilometers, they provide a mechanism for meadow recovery (Tol et al., 2017). Herbivores reduce the accumulation of organic matter and nutrients by consuming seagrass, reducing the risk of factors such as hypoxia and diseases that cause seagrass die-off (Jackson et al., 2001; Christianen et al., 2012). Megaherbivore grazing also increases microbial nutrient cycling in seagrass sediments (Perry and Dennison, 1999).

Tropical seagrass responses to grazing pressure are dependent upon the size and densities of herbivores present. Some overall patterns can be observed for tropical meadows, and are summarized in **Figure 1**, but variability between meadows still occurs due to meadow characteristics and differences in spatial or temporal scale of studies. There is variability both between seagrass species and due to differences between study locations. Studies within the same location have also produced differing results (Myers and Heck, 2013; Mutchler and Hoffman, 2017). Seascape configuration and the proximity of other habitats can have an impact on seagrass meadow fauna and meadows in proximity to other habitats can have increased herbivory (Valentine et al., 2008; Swindells et al., 2017).

## Interactions Among Herbivore Functional Groups

Grazing by one herbivore group can change seagrass meadows as habitats, in ways that affect other herbivores. Heavy grazing by megaherbivores can diminish the available habitat for mesograzers, and the suitability of habitat for macroherbivores. The consumption of epiphytes by mesograzers may be positive for herbivores that consume seagrass directly, due to increased seagrass growth. The removal of epiphytes, however, might also negatively affect larger herbivores, many of which gain nutrition from the epiphytic algae, either instead of, or in addition to, seagrass itself (Marco-Méndez et al., 2012, 2015). Larger herbivores may inadvertently consume mesograzers while feeding on the seagrass they live among (Marsh et al., 2011). Interactions also occur within grazer groups. Herbivory by fish can increase predation risk to sea urchins by reducing habitat complexity and making them more visible (Pagès et al., 2012). Grazing can cause changes to seagrass habitat complexity, which can affect where fish choose to feed, with higher fish herbivory in more complex sites (Unsworth et al., 2007). Chemical changes in seagrass tissue composition caused by herbivory can be beneficial to herbivores. Nitrogen content can increase in response to herbivory, making the seagrass more nutritionally rich (Aragones et al., 2006). However, these changes can be negative, with reductions in starch and increases in fiber (Aragones et al., 2006; Jimenez-Ramos et al., 2017). Phenolic compounds defend seagrasses against herbivores by changing seagrass palatability, and their production shows differing responses to grazing pressure, exhibiting both increases (Martínez-Crego et al., 2015) and decreases (Vergés et al., 2008).

## Herbivory as an Agent of Ecosystem Service Change

**FIGURE 2:**
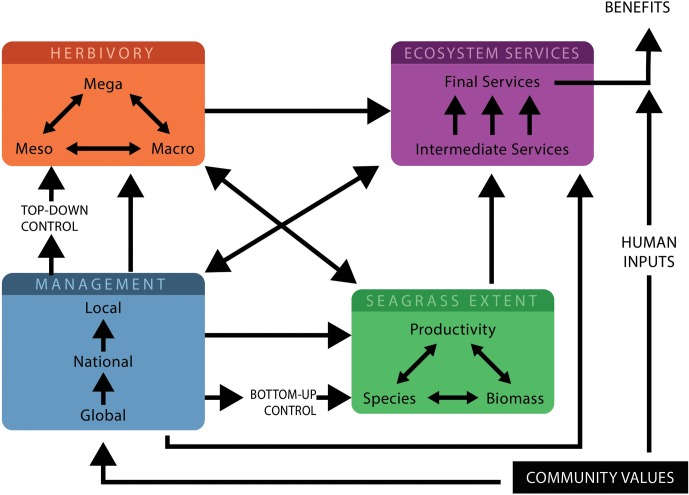
The interactions in a seagrass-herbivore system for managers and researchers to consider, to maintain a balanced system. Different herbivores interact with each other to modify seagrass properties and ecosystem services which depend on herbivore numbers, herbivore numbers are determined by top–down controls. Management measures can be dictated by global policy and can be national or local/community based. These measures which control human activities will influence seagrass properties, herbivores and ecosystem services and will in turn be influenced by the relative importance of the various community values and important ecosystem services. Seagrass extent is determined by productivity, species and biomass as well as bottom–up controls, this influences both the ecosystem services provided and the number of herbivores feeding. Ecosystem services are influenced by seagrass extent, herbivores and management measures and require human inputs for benefits to be realized.

Grazing intensity and type (e.g., shredding, cropping, or excavating) structures seagrass meadows and influences the level and type of ecosystem services provided. If the intensity of herbivory is moderate, productivity may increase, resulting in more nutrient uptake by the seagrass (Christianen et al., 2012). Grazing that leads to loss of biomass and reductions in shoot height may alter intermediate services provided by seagrasses. Including potential reductions in a meadow’s capacity to: act as a nursery habitat (Heck et al., 2003; Nagelkerken et al., 2004; Sheaves et al., 2014), trap sediment (De Boer, 2007), and sequester carbon (Lavery et al., 2013; Atwood et al., 2015). At very high levels of herbivory, seagrass productivity may be unable to keep pace with removal rates and the meadow could collapse, as shown in **Figure 1** (Fourqurean et al., 2010; Christianen et al., 2014; Heithaus et al., 2014). In this case, ecosystem services would cease to be delivered, and stored biomass or sediment carbon could be released back into the environment (Fourqurean et al., 2012; Macreadie et al., 2015). Meadow loss on a large scale also results in mortality and changes in fecundity in seagrass-dependent herbivore populations (Meager and Limpus, 2014; Fuentes et al., 2016). How plant–herbivore interactions change ecosystem services depends on location, season, habitat type, seagrass species and the herbivore community composition. Some services are more valuable in certain locations; e.g., the amount of carbon sequestered by seagrasses depends on seagrass species and the environmental context in which the meadow occurs (Lavery et al., 2013; Serrano et al., 2016). Other factors that influence seagrass and herbivores will also change ecosystem service delivery by mediating plant–herbivore interactions as shown in **Figure 2**. Bottom–up anthropogenic stressors and environmental conditions (e.g., light and nutrient levels) can influence seagrass structure, and the top–down influence of predator presence determines where herbivores are more likely to feed (Atwood et al., 2015; Bessey et al., 2016). The response of services to anthropogenic or abiotic disturbance is dependent on the type and intensity of the stressor, and can be context-dependent (Díaz et al., 2007b). Sometimes the impact of herbivores on seagrass ecosystem service delivery is unexpected; for example even when meadows are heavily grazed, the below-ground biomass can still provide an important coastal protection service (Christianen et al., 2013).

By altering the species composition in seagrass meadows and creating disturbance, herbivores can change biodiversity in seagrass communities. Because seagrass is disturbed by grazing activity, both colonizing and more persistent species will be present, increasing seagrass diversity (**Figure 1**) (Rasheed, 2004; Kelkar et al., 2013). Terrestrial ecosystems with more plant species provide higher levels of ecosystem services (Gamfeldt et al., 2013). Increases in diversity are associated with increased provision of ecosystem services and greater multi-functionality of systems, attributed to greater interspecific niche complementarity (Cardinale et al., 2012; Gamfeldt et al., 2013; Lefcheck et al., 2015); however, the high level of complexity in diverse communities may also lead to a greater number of negative interactions and trade-offs (Duncan et al., 2015). The dominant plant species in ecological communities can be the predominant drivers of ecosystem functioning (mass ratio: cf. terrestrial grassland examples: Grime, 1998; Díaz et al., 2007a). The identity of dominant seagrass species, and their interactions with herbivore groups, may also play a role alongside, or instead of, high functional diversity to influence seagrass ecosystem service delivery.

There are links between intermediate and final seagrass ecosystem services, some of which are well-established, such as changes in seagrass primary production and mesograzer removal of epiphytes mitigating nutrient pollution (Christianen et al., 2012). Yet for others, the relationship is unclear. Ecosystem services and human well-being are linked, but the relationship is neither consistent nor linear, so it is difficult to predict how well-being outcomes respond to pressure (Baker et al., 2015).

Herbivores themselves are also important for the ecosystem services delivered by a seagrass meadow. The ecosystem service benefits of tourism, hunting, fishing, and cultural values depend explicitly on the presence of herbivores (Butler et al., 2012; Cullen-Unsworth et al., 2014). Cultural ecosystem services provided by seagrass meadows are important, but they are understudied, difficult to quantify and are rarely incorporated into management (Garcia Rodrigues et al., 2017; Ruiz-Frau et al., 2017). Understanding cultural services in the tropics is important, as spiritual and religious values of seagrasses are significant and qualitative information on this is available (De La Torre-Castro and Rönnbäck, 2004; Cullen-Unsworth et al., 2014). Dugongs and green turtles have been referred to as cultural keystone species for communities in the tropics (Butler et al., 2012). Some cultural services such as education, tourism, and research require human inputs for benefits to be realized. Others, such as religious, spiritual and bequest value, can be viewed as final services as they rely on a functioning seagrass-herbivore system.

In **Figure 1** we summarize how seagrass and herbivore interactions manifest to affect the delivery of key ecosystem services. Our model suggests that at low levels of herbivory, the system is seagrass dominated, characterized by high seagrass biomass and moderate levels of productivity and diversity. As herbivory increases, the system moves toward a balanced state where productivity increases in response to herbivory and productivity-associated ecosystem services’ (i.e., carbon sequestration and storage, nutrient uptake leading to improved water quality) delivery increases. In this system, we hypothesize the diversity of both seagrass and herbivore assemblages are generally at their highest and the biomass of both seagrass and herbivores are maintained at moderate levels. As herbivory increases further, seagrass biomass, diversity and productivity decreases and most ecosystem services’ delivery reduces before the meadow becomes overgrazed and collapses, at which point ecosystem services’ delivery ceases. In the model cultural ecosystem services’ delivery has not been quantified, while it is recognized as being important and may be influenced by herbivory, responses are likely to be highly variable and are not well-understood (Díaz et al., 2006; Garcia Rodrigues et al., 2017). **Figure 1** hypothesizes that a balanced system will maximize the broadest range of ecosystem services. While some individual services may peak in either seagrass dominated systems (e.g., nursery habitat and sediment trapping) and others in herbivore dominated systems (e.g., tourism), the presence of intermediate levels of biomass and higher diversity of both seagrasses and herbivores ensures that the greatest number of services will be provided by this balanced state.

## Ecosystem Service Interactions

Ecosystem services can interact with each other as they respond to pressure. Where people and seagrass interact, there are many trade-offs and synergies in service delivery (Arthur et al., 2013; Bas Ventin et al., 2015; Garcia Rodrigues et al., 2017). For example, an increase in both large herbivore numbers and seagrass biomass beyond a threshold value is unlikely, so services associated with herbivores will increase, while those associated with seagrass habitat decrease, resulting in a trade-off. Synergies may also occur but are poorly understood in relation to herbivore pressure.

Understanding interactions and trade-offs in a seagrass meadow and making them explicit is imperative for predicting future changes in delivery, trade-offs, and outcomes of management decisions (Mouchet et al., 2014). Even well-intentioned measures can have unintended consequences, or perverse outcomes. Implementing no-take marine protected areas (MPAs) can result in higher local intensities of fish herbivory and consumption of seagrass production (Alcoverro and Mariani, 2004). MPAs designed to protect green turtles, can cause aggregations that overgraze the seagrass and lead to meadow collapse (Christianen et al., 2014). This effect may be exacerbated if declines in top predators that control green turtles allow green turtle populations to exceed historical numbers (Burkholder et al., 2013; Heithaus et al., 2014). However, green turtles are threatened in tropical seagrass areas and are at high risk of climate change-associated declines (Fuentes et al., 2011), creating a trade-off in potential management priorities.

## A Pathway for Effective Management of Herbivores, Seagrasses and Their Services

Conservation practitioners and managers can use many legislative instruments to protect seagrasses and their herbivores. These can be global, national, or local in scale and with different objectives; i.e., to protect a certain area, a given species or ecosystem type. This range in scale and scope mean that differing pieces of legislation do not always work well together. Management actions can have local consequences, or affect services that have global implications, such as carbon sequestration. To conserve tropical seagrasses and the services they provide, a holistic approach is needed and, to avoid any unconscious bias influencing decisions, weightings should be made explicit. With an awareness of all the interactions at play, we can understand the impact of management decisions and how best to achieve objectives sustainably and across different scales (Arkema et al., 2015).

Management actions will generally prioritize a given set of ecosystem services, which will then require a different seagrass community structure as shown in **Figure 1**, however, the variation in seagrass properties and associated services will depend on the types and numbers of herbivores present. Simultaneous multiple benefits could potentially be maximized with minimal impact on the desired set of ecosystem services, by managing for a balanced system (**Figure 1**). Management decisions that shift systems to either seagrass or herbivore dominated are likely to produce trade-offs across a range of services. Where management decisions are skewed away from the maintenance of a balanced system, undesirable outcomes for some ecosystem services are possible and, in the worst-case scenario, complete collapse can occur. For example, if services such as sediment trapping are a priority, managers may wish to aim more toward a seagrass-dominated state, however trade-offs will occur in some other services as a result and should be factored into management decisions. Managing for the balanced system will likely maximize biodiversity benefits, which are a global-scale target. Despite this, a balanced system may not reflect the community desires for seagrass ecosystem service priorities, a critical component in any management framework (**Figure 2**). While we contend a balanced system is likely to be the most sustainable in the long term, managing for other states is possible and we provide the framework for understanding the consequences of these through the interactions of management decisions with seagrasses, herbivores and their controls in **Figure 2**. The states shown in **Figure 1** are not separate groups, but are on a continuum such that managers can aim toward a system which is more seagrass or herbivore dominated depending on their ecosystem service priorities and the local community priorities.

Knowledge of the complex interactions between herbivores, seagrasses and delivery of ecosystem services is required to achieve balanced systems or other desired management outcomes and the consequences of these. **Figure 2** highlights the critical precursors and major pathways and interactions to consider in the tropics for effective management of seagrass-herbivore interactions. This figure illustrates how interactions between herbivores can alter seagrass properties and modify ecosystem service delivery, but also illustrates the top–down and bottom–up factors and management pathways which can influence ecosystem services. Where possible, it is desirable to assess the relative importance of interactions and to incorporate them into management processes. Predicting all interaction outcomes is impossible, but understanding patterns in interaction outcomes can provide guidance to managers (Côté et al., 2016). Conserving seagrass meadow fauna in the tropics requires targeted management, especially given the overexploitation of these animals, and of herbivores in particular, with many populations still vulnerable or endangered (Jackson et al., 2001; Unsworth and Cullen, 2010).

To design effective, balanced management, or an understanding of the consequences of management decisions directed in favor of a particular service, an awareness of the elements detailed in **Figure 2** is required. These involve:

(1) understanding the desirable management outcome and community values and making their perceived relative importance explicit;
(2) evaluating potential undesirable outcomes for the environment and local community including possible trade-offs;
(3) identifying top–down and bottom–up controls in the system that can be manipulated by management actions; and
(4) monitoring seagrass ecosystem services and adapting management plans accordingly.

Enabling sustainable management of tropical seagrass ecosystem services requires critical research gaps on how plant–herbivore interactions shape ecosystem service delivery to be addressed. In particular, research is needed to understand how:

(1) different herbivore size classes interact to structure seagrass meadows?
(2) ecosystem services interact in response to herbivory pressure?
(3) the local community values the relative importance of the trade-offs?
(4) management actions help to realize benefits of incorporating community values into actions, especially with regard to cultural services.

## Conclusion

We contend that a balanced system that promotes diversity of plant and herbivore assemblages is likely to be desirable for sustaining and maintaining delivery of multiple seagrass ecosystem services as shown in **Figure 1**. Seagrass communities are complex systems with potential for poor outcomes if we fail to understand the interactions, trade-offs and unintended consequences that can occur. **Figure 2** highlights the pathways for managers to be aware of, and to act through, to maximize opportunities to achieve desired outcomes for seagrasses, herbivores and ecosystem services. Seagrass ecosystem services in tropical meadows are poorly understood (Ruiz-Frau et al., 2017), and there are research gaps in relation to herbivore activity that need to be addressed, in addition to the more general seagrass ecosystem services research gaps identified in Nordlund et al. (2017). A focus on cultural ecosystem services will allow a more informative valuation of the social, economic and ecological benefits of tropical seagrass systems. It is clear that herbivory is a major structuring influence in tropical seagrass systems and needs to be considered alongside traditional “seagrass only” focused assessments for effective management of these critical habitats and their services. Many of our conclusions are based on hypothetical relationships derived from theory or temperate seagrass systems. Nevertheless, as well as a guide to management decisions based on current knowledge, our framework is useful to show critical areas for future research.

## Author Contributions

ALS, PHY, MAR, PIM, and CD conceived the main concept of the manuscript. All authors participated in a workshop to discuss and develop the themes of the perspective article. ALS wrote the manuscript. All authors reviewed, revised, and approved the final manuscript.

## Conflict of Interest Statement

The authors declare that the research was conducted in the absence of any commercial or financial relationships that could be construed as a potential conflict of interest. The handling Editor is currently co-organizing a Research Topic with one of the author PIM, and confirms the absence of any other collaboration.
